# Human–Robot Cooperative Strength Training Based on Robust Admittance Control Strategy

**DOI:** 10.3390/s22207746

**Published:** 2022-10-12

**Authors:** Musong Lin, Hongbo Wang, Congliang Yang, Wenjie Liu, Jianye Niu, Luige Vladareanu

**Affiliations:** 1 Hebei Provincial Key Laboratory of Parallel Robot and Mechatronic System, Yanshan University, Qinhuangdao 066004, China; 2 Academy for Engineering & Technology, Fudan University, Shanghai 200433, China; 3 Shanghai Clinical Research Center for Aging and Medicine, Shanghai 200040, China; 4 Key Laboratory of Advanced Forging & Stamping Technology and Science of Ministry of Education, Yanshan University, Qinhuangdao 066004, China; 5 Robotics and Mechatronics Department, Institute of Solid Mechanics of Romanian Academy, 010141 Bucharest, Romania

**Keywords:** rehabilitation robot, human–robot interaction, admittance control, robust control, active strength training

## Abstract

A stroke is a common disease that can easily lead to lower limb motor dysfunction in the elderly. Stroke survivors can effectively train muscle strength through leg flexion and extension training. However, available lower limb rehabilitation robots ignore the knee soft tissue protection of the elderly in training. This paper proposes a human–robot cooperative lower limb active strength training based on a robust admittance control strategy. The stiffness change law of the admittance model is designed based on the biomechanics of knee joints, and it can guide the user to make force correctly and reduce the stress on the joint soft tissue. The controller will adjust the model stiffness in real-time according to the knee joint angle and then indirectly control the exertion force of users. This control strategy not only can avoid excessive compressive force on the joint soft tissue but also can enhance the stimulation of quadriceps femoris muscles. Moreover, a dual input robust control is proposed to improve the tracking performance under the disturbance caused by model uncertainty, interaction force and external noise. Experiments about the controller performance and the training feasibility were conducted with eight stroke survivors. Results show that the designed controller can effectively influence the interaction force; it can reduce the possibility of joint soft tissue injury. The robot also has a good tracking performance under disturbances. This control strategy also can enhance the stimulation of quadriceps femoris muscles, which is proved by measuring the muscle electrical signal and interaction force. Human–robot cooperative strength training is a feasible method for training lower limb muscles with the knee soft tissue protection mechanism.

## 1. Introduction

The independent walking ability of the elderly is the basic premise to ensure the quality of life [1]. However, limb weakness increases with age and the impact of cardiovascular disease often leads to physical disability in the elderly [2]. According to statistics, there are more than millions new incident stroke cases worldly in every year, and there is a high probability of losing walk ability among the survivors [3,4]. Facing such a large number of disabled people, more rehabilitation physicians and rehabilitation training equipment are needed to help them regain lower limb strength, stand up again and return to society [5,6]. As a new type of intelligent medical robot, rehabilitation robot can effectively improve limb disabilities caused by aging or sequela and their therapeutic effect has been proved by many clinical experiments [7,8,9].

Muscle weakness is well established as the primary impairment that affects walking after stroke, and strength training can effectively promote the recovery of muscle strength [10,11]. The effectiveness of strength training has also been proven by some resistance training [12,13]. The quadriceps femoris is the biggest human skeletal muscle at the front of the thigh, and it plays a vital role in extending the knee, flexing the hip and maintaining an upright position. Leg flexion and extension is a strength training exercise that can effectively enhance the quadriceps, so the research of related equipment has also attracted much attention. The American Harley Company proposed a rehabilitation device X-10, which uses variable pressure technology to reduce the pain in the patient’s treatment and improve the patient’s joint mobility [14]; Another similar device is a sitting rehabilitation device developed by King Wangut University of Technology, which is suitable for home training but only has one free rotation and a small range of motion [15]. They all belong to the same type of rehabilitation equipment using a moving platform. They send the terminal force and joint torque into the control feedback loop to ensure the safety of training respectively, but the training effect on the hip joint is not obvious. The University of Tsukuba and a partner company developed an exoskeleton robot called HAL, which can directly provide active or passive lower limb flexion and extension training to bedridden patients. The range of motion and the walking ability of the patients is improved after training, but the strength of the quadriceps femoris does not change significantly [16,17]. Italy and Poland developed a new 3-DOF bionic exoskeleton, which can be used for rehabilitation after joint surgery, ligament, and cartilage injuries [18]. 

In robot-assist active training fields, the robot needs to be able to extract the patient’s motion intention according to the interaction information and assist the patient to complete the training action. The effectiveness of impedance control and force-position hybrid control have been verified on the rehabilitation machine LOKOMAT, and these methods have improved the interactivity of human–machine cooperative training [19]. Wu et al. developed an admittance control strategy induces the active participation of patients [20]; an optimization method based on admittance control was proposed to compensate the weight and friction of the exoskeleton [21]. A lower rehabilitation robot called LOPES II allows different active training intensities through admittance control [22]. Impedance controllers are also applied in robot-assist active training for joint or lower limb rehabilitation [23,24]. Some researchers use sEMG (surface electromyography) or EEG (electroencephalography) signals for guiding rehabilitation robots to complete active training [25,26,27]. Courtney et al developed an algorithm for adjusting functional electrical stimulation to help patients taking active training [28]. 

Including the research mentioned above and other we can find, none have mentioned the protection of the knee soft tissue. However, the physiological functions of the elderly gradually degenerate, and soft tissues such as the meniscus, cartilage and ligaments are relatively fragile [29,30]. For the main user groups of rehabilitation therapy, it is necessary to avoid damage to their joint soft tissues during rehabilitation strength training. The National Strength and Conditioning Association has studied knee joint biomechanics during the human squat and pointed out the conclusion. That is, the tibiofemoral compressive force will peak at 130 degrees of knee flexion, and the menisci and articular cartilage bear significant amounts of stress [31]. Soft tissue such as ligaments are at great risk of injury at this moment [32]. Patellofemoral compressive force, tibiofemoral compressive force and tibiofemoral shear force will gradually decrease with knee extension, while quadriceps muscle activity will peak at approximately 80 to 90 degrees of knee flexion and remain relatively stable thereafter [33,34]. In the human–machine cooperative leg flexion and extension training, it is necessary to timely control the interaction force depending on the knee joint angle in order to reduce the possibility of joint soft tissue injury. 

In this paper, a human–machine cooperative leg flexion and extension training based on a robust admittance control strategy is proposed, which fully considers the protection of knee soft tissue based on biomechanics. The performance device is the sitting and lying lower limb rehabilitation robot (LLR-II) developed by our team. In this training, LLR-II responds according to the interaction force and assists the patient performs a full lower limb flexion and extension similar to a leg press. Compared with single knee flexion and extension training, this training can maintain and improve the mobility of each joint of the lower limbs, and can effectively exercise the muscles of the hips, knees and ankles. Firstly, according to the biomechanics of the knee joint, the change law of the stiffness of the main admittance model is designed, and the flexibility of the training is increased by the subsidiary admittance control. The controller will adjust the model stiffness according to the joint angle during the training, and it could avoid excessive compressive force on the soft tissue and increase the stimulation of the quadriceps. Then, the joint tracking performance is improved by two-input robust motion control by compensating the motion control disturbances caused by model uncertainty, interactive forces, and external noise. Finally, the testing experiment of this human–machine cooperative leg flexion and extension training is conducted.

## 2. LLR-II Rehabilitation Robot

**Figure 1:**
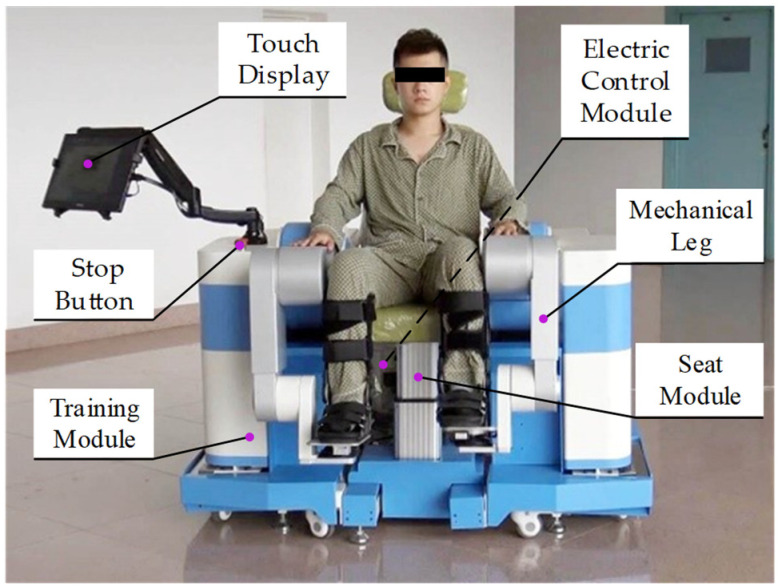
Structure of LLR-II.

The LLR-II is an intelligent robotic system that can intervene early and provide a variety of rehabilitation training and more details can be found in our published papers [35,36]. LLR-II can be divided into four modules which include two symmetrical training modules, a seat module and an electric control module, as shown in Figure 1. The LLR-II is assembled by connecting the underframe of each module and each module can be moved independently for installation and transportation. The right training module is equipped with a touch display and an emergency stop button and the width between the two training modules can be adjusted according to the user’s body shape. The height of the seat module is adjustable and it can help medical staff transfer patients. In addition, in order to adapt to different people, the length of the upper and lower mechanical legs can be adjusted through the internal electric linear actuator.

### 2.1. Structural Design of LLR-II

**Figure 2:**
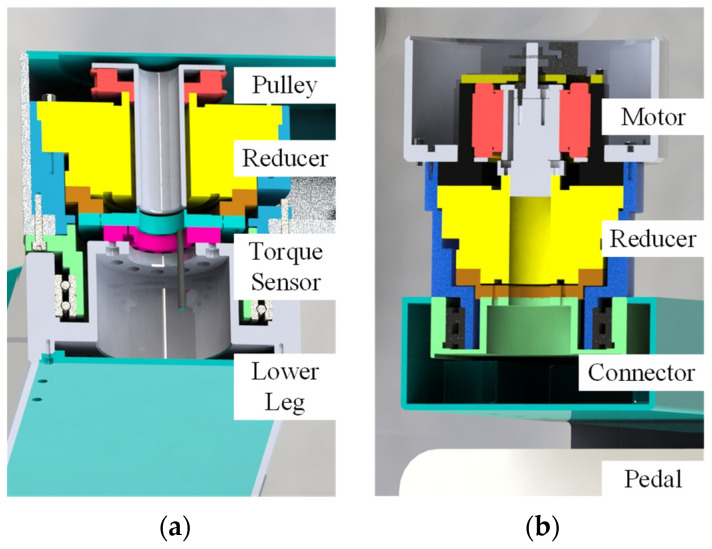
Section view of joint drivetrain: (**a**) knee joint and (**b**) ankle joint.

The mechanical leg of LLR-II is a three joint series mechanical mechanism, and the three joints correspond to the hip, knee and ankle joints of the human body, respectively. Its joint drive train is composed of flange structures, as shown in Figure 2. The high torque motors of the hip and knee joints adjust the fixed positions through timing belts, which are located at the bottom of the training module and the rear end of the mechanical leg respectively. Hip and knee joint transmission structures are similar, and both of them are consist of a synchronous pulley, a reducer and a torque sensor (Figure 2a). The ankle joint equips with a frameless motor, and the integration of the ankle joint is effectively improved by directly connecting the motor and the reducer (Figure 2b).

**Figure 3:**
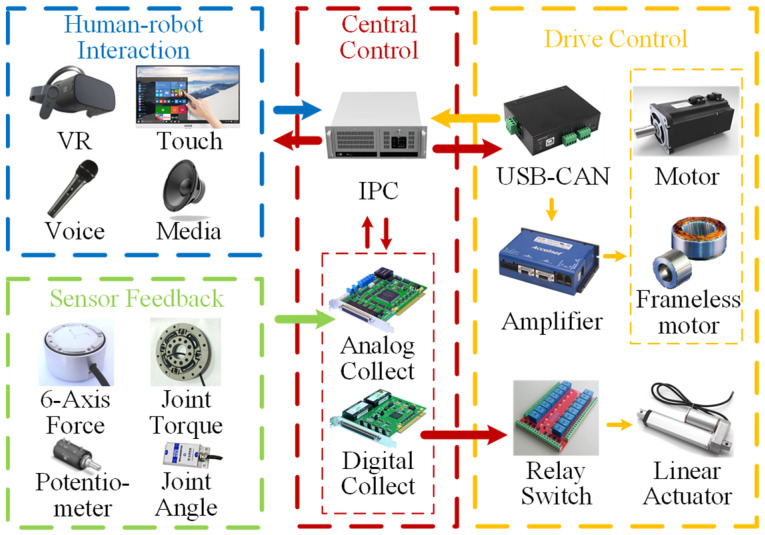
Electric control system. The arrow represents the direction of information transmission.

The electric control system of LLR-II can be divided into four parts as follows: central control section, drive control section, sensor feedback section and human–robot interaction section (Figure 3). The central control section mainly includes the host computer and related data acquisition equipment, which is responsible for the advanced operations and coordinates other parts. The drive control section is mainly composed of the joint motor, the electric linear actuator and the related communication control equipment. The sensor feedback section mainly includes the torque sensor, the angle sensor of the joint, the six-dimensional force sensor and the potentiometer. The interaction operation is mainly realized through a touch display screen. In addition, the LLR-II also has multimedia functions such as virtual reality and voice control.

### 2.2. Mechanical Leg Model Analysis

**Figure 4:**
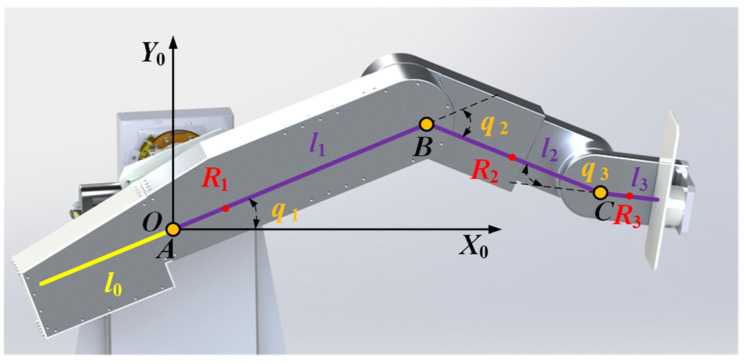
Mechanism model of the mechanical leg.

The mechanical leg of LLR-II is a series manipulator working in the sagittal plane, and its physical model can be simplified as a 3R structure, as shown in Figure 4.

Establish a global coordinate system {*O*-*X*_0_*Y*_0_*Z*_0_} at the hip joint rotation center point *A*. *B* and *C* represent the rotation centers of the knee and ankle joints respectively. *q*_1_, *q*_2_ and *q*_3_ are the joint variables of the three rotating joints, *l*_1_, *l*_2_ and *l*_3_ respectively represent the distance between the rotating joints, *l*_0_ represents the distance between the counterweight mess center and the hip rotating joint; *R*_1_, *R*_2_ and *R*_3_ represent the distance between the link mass center and the rotation center, respectively. The kinematic model of LLR-II is the same as the standard 3R mechanism, and its kinematics forward and inverse solutions can be calculated by the D-H method and geometric method. The results are shown in Equations (1) and (2) below:

(1) $$T=\left[\begin{array}{cccc} n_{x} & o_{x} & a_{x} & p_{x}\\ n_{y} & o_{y} & a_{y} & p_{y}\\ n_{z} & o_{z} & a_{z} & p_{z}\\ 0 & 0 & 0 & 1 \end{array}\right]=\left[\begin{array}{cccc} \cos q_{123} & -\sin q_{123} & 0 & l_{3}\cos q_{123}+l_{2}\cos q_{12}+l_{1}\cos q_{1}\\ \sin q_{123} & \cos q_{123} & 0 & l_{3}\sin q_{123}+l_{2}\sin q_{12}+l_{1}\sin q_{1}\\ 0 & 0 & 1 & 0\\ 0 & 0 & 0 & 1 \end{array}\right],\;$$

(2) $$\begin{array}{l} q_{1}=\mathrm{Atan}2(K,\;\pm \sqrt{1-K^{2}})-A\tan2(A,\;B)\\ q_{2}=\mathrm{Atan}2(B-l_{1}\sin q_{1},\;A-l_{1}\cos q_{1})-q_{1}\\ q_{3}=\mathrm{Atan}2(n_{y},\;n_{x})-q_{1}-q_{2} \end{array},$$

where

$$\begin{array}{l} A=p_{x}-l_{3}n_{x}\\ B=p_{y}-l_{3}n_{y}\\ K=\frac{A^{2}+B^{2}+l_{1}^{2}-l_{2}^{2}}{2l_{1}\sqrt{A^{2}+B^{2}}}\\ q_{1\ldots i}=q_{1}+\ldots +q_{i} \end{array}.$$

The dynamic equation of the mechanism can be obtained through the Lagrangian equation, and the controlled system model of the robot can be obtained as follows:

(3) $$M(q)\ddot{q}+C(q,\dot{q})\dot{q}+g(q)+J^{T}(q)F=u,$$

where M(q) is a diagonal matrix consisting of the inertia matrix and drive train inertia. $C(q,\dot{q})$ represents the matrix of Coriolis and centrifugal forces and g(q) represents the gravitational vector. $\ddot{q}$, $\dot{q}$ and q are the joint acceleration, velocity and position vectors. J(q) is the Jacobian matrix of the mechanism and F represents the human–robot interaction force. u is the control input vector.

Unlike the standard 3R structure, this mechanism has a counterweight used for lightening the motor load, as the yellow line shown in Figure 4. The leg length adjustment function is adjusting the position of the rotation center point *A* and it means that the relative position of the link mass center point *R*_1_ in the global coordinate system will change under the influence of the counterweight and the leg length change. The Lagrangian quantity change caused by the mass center position change will exacerbate the system uncertainty in the dynamic control. Therefore, it is necessary to calculate the mass center position of the link *l*_1_, as shown in Equation (4) below:

(4) $$R_{1}=\frac{m(l_{1}-l_{0})-2m_{0}l_{0}}{2m+2m_{0}},\;$$

where *m*_0_ is the mass of the counterweight and *m* is the mass of the first link (without counterweight). In Lagrangian dynamics, the change of the mass center position will directly change the translational kinetic energy and gravitational potential energy of the first link, and the moment of inertia in the angular kinetic energy term also needs to be recalculated according to Equation (4) after length adjustment.

## 3. Robust Admittance Control Strategy

**Figure 5:**
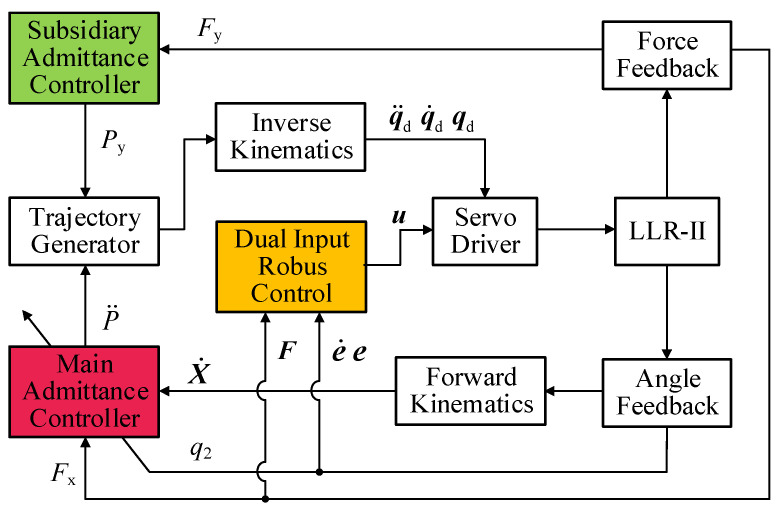
Admittance robust control block diagram.

The control strategy of the rehabilitation robot is different from the general industrial robot; it needs to fully consider human–robot interactions to ensure the safety of patients. Rehabilitation robots should be able to respond to different levels of interaction and maximize the movement potential of patients. Biomechanical research has shown that pushing force should be avoided when the knee is flexed at a wide angle, and there is also an efficient training range for the quadriceps. In addition, due to the large interactive force of the active training, it has high requirements for the robustness of the control algorithm. Therefore, a robust admittance control strategy for lower limb strength training is proposed, which combines robust control and admittance control. The strategy block diagram is shown in Figure 5. This strategy indirectly controls the user’s force through variable stiffness admittance control, and it can avoid excessive compressive force on the joint soft tissue and increase muscle group stimulation. Dual input robust control adds an error compensation term that can be used for compensating force interference, and it improves the tracking performance of the machine joints.

### 3.1. Variable Stiffness Admittance Control

**Figure 6:**
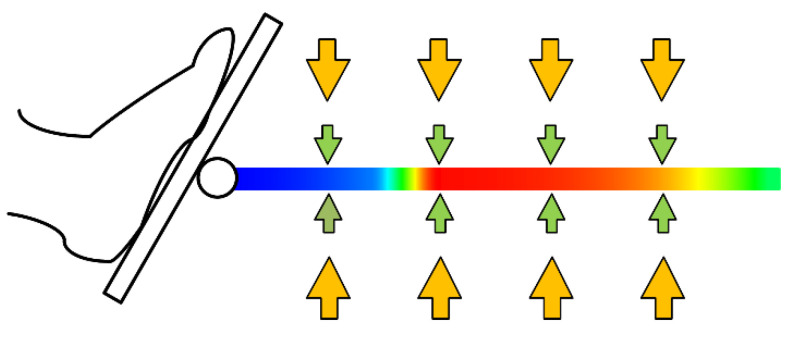
Working schematic diagram of admittance controller.

Admittance control is a control strategy that describes the relationship between force and motion through a spring damping model, and both admittance control and impedance control use the same model. The input and output of admittance control are force and position, respectively. The end force of the series robot can be easily obtained by force sensors, so this method is often used in human–robot interactions. The admittance control strategy proposed in this paper includes two control laws, and its function is shown in Figure 6. The effect of the main control law is changing the model stiffness according to the knee joint angle; it can protect the knee joint and increase the stimulation of the quadriceps muscle (the stiffness change is plotted on a trajectory with color mapping in Figure 6, the bright part indicates high stiffness). The subsidiary control law allows a small deviation of the training trajectory, which increases the flexibility of the training action. When the user’s vertical force will lead to a large deviation of the trajectory, the subsidiary control law will ensure the trajectory by resisting the user’s force (as arrows shown in Figure 6).

The input of the subsidiary admittance control is the interaction force *F_y_*(s) in the vertical direction, and the output is the end position *P_y_*(s) in the vertical direction. Its transfer function is shown in Equation (5):

(5) $$G(s)=\frac{P_{y}(s)}{F_{y}(s)}=\frac{1}{M_{\mathrm{dy}}s^{2}+B_{\mathrm{dy}}s+K_{\mathrm{dy}}},\;$$

where *M*_dy_, *B*_dy_ and *K*_dy_ represent inertia, damping and stiffness. The system will compare the expected position with the set offset threshold. If output exceeds the set allowable offset, the excess part will be limited. Two outputs of the subsidiary control and main control will be sent to the trajectory generator, and the generator will obtain the actual end position based on vector calculation before inverse kinematics. With smaller model parameters, the compliant and constrained training trajectory can be achieved.

The model of main admittance control is a second-order model with varying stiffness along the motion trajectory and the output $\ddot{P}$ is the desired end acceleration. When receiving an interaction force exceeding the threshold, the end of the machine will accelerate. When the interaction force is deficient, the end of the machine will decelerate to stop according to the admittance parameters. Its control law is designed as follows:

(6) $$M_{\mathrm{dx}}\ddot{P}+B_{\mathrm{dx}}{\left\|\dot{X}\right\|}_{2}+K_{\mathrm{var}}(q_{2})D=F_{x},\;$$

where *F*_x_ is the extracted effective interaction force; *D* is a constant with the same dimension as the end position; $\dot{X}$ represents the end velocity vector of the robot. *M*_dx_ and *B*_dx_ are the inertia and damping of the model and the model stiffness ***K***_var_ is a piecewise function of the knee joint angle, designed as follows:

$$K_{\mathrm{var}}=\left\{ \begin{array}{l} k_{1}+\frac{(k_{2}-k_{1}){\left[L(q_{2})-L_{0}\right]}^{2}}{{\left[L_{1}-L_{0}\right]}^{2}}\;\;\;\;\;\;\;\;\;\;-120^{{}^{\circ}}\leq q_{2}<-100^{{}^{\circ}}\\ \frac{k_{2}+k_{3}}{2}-\frac{k_{3}-k_{2}}{2}\cos(\pi \frac{L(q_{2})-L_{1}}{L_{2}-L_{1}})\;\;\;\;\;\;-100^{{}^{\circ}}\leq q_{2}<-90^{{}^{\circ}}\\ k_{3}-k_{4}\exp\left[k_{5}\frac{L(q_{2})-L}{L-L_{2}}\right]\;\;\;\;\;\;\;\;-90^{{}^{\circ}}\leq q_{2}\leq 0^{{}^{\circ}} \end{array}\right..$$

*L*(*q*_2_) is a function of the knee joint angle and leg length; it represents the end position of the robot. *L*_0_, *L*_1_ and *L*_2_ represent the end position scalars of the robot when the knee joint is at −120°, −100° and −90°; *L* represents the total length of the training trajectory. The constant coefficients *k*_i_ (i = 1, 2 …) are all parameters of this function and the amplitude of stiffness can be adjusted by changing these parameters.

In this training process, the motion range of the knee joint is −120° to 0°, which covers 80% normal motion range of the human body. The purpose of this design is to stretch the muscles of the knee joint and maintain joint mobility. In addition, the controller will adjust the model stiffness in real-time according to the knee joint, and this can protect the knee soft tissue and increase the stimulation effect on the quadriceps. At the beginning of the training (120° of knee flexion), the model stiffness is set at a low level. This is because, in this angle range, it will put greater stress on the knee soft tissue when the leg extends with resistance forces. Training in this situation for a long time might cause damage to the knee joint. When the knee joint is flexed to 100°, the model stiffness begins to rise rapidly. When the knee joint is flexed to 90°, the stiffness reaches the highest level, which marks the training entering the strong stimulation phase. At this stage, the force of the lower limbs mainly depends on the contraction of the quadriceps femoris, and the training effect can be improved by correctly exerting force in this stage. In the final stage, the stiffness decreases slowly with a negative exponential trend. Considering the lower limb is not easy to exert force when it is close to full extension, this design can extend the stimulation movement and ensure that the user can complete the leg extension.

### 3.2. Dual Input Robust Control

Robot dynamics control needs to solve the tracking error problem caused by external disturbance or model inaccuracy. For most of the series robots, the model inaccuracy mainly comes from the uncertainty of the dynamic parameters (the deviation between the theoretical reference model and the actual model). This uncertainty is generally changeless and can be reduced by optimizing parameters through classical algorithms. The model structure of the LLR-II is rather special as the first link mass center position becomes a variable under the influence of the counterweight structure and the length adjustment. Therefore, the parameters of the dynamic model will change greatly after the mechanical leg length adjustment because the mass center change will lead to the change of Lagrangian variables. That is to say, the parameter uncertainty of the LLR-II model is also a variable. Although the reference model will be updated according to Equation (4), there is still a deviation from the actual model. Adding to the influence of the large fluctuation interaction force, common classical algorithms cannot adapt to such variable parameter systems.

This paper proposes a dual input robust control considering the s interactive force effect, and it is used for reducing the influence of model uncertainty, noise interference and the impact of interactive forces on machine tracking performance. The design control law is as follows:

(7) $$u=\hat{M}(q)a+\hat{C}(q,\dot{q})v+\hat{g}(q)+{\hat{J}}^{T}(q)\hat{F}-Kr,$$

where $\hat{M}$, $\hat{C}$, $\hat{g}$ and $\hat{J}$ are estimated values defined by the corresponding symbols (theoretical reference value); ***K*** and ***Λ*** are two constant positive gain matrices; ***v***, ***a*** and ***r*** are defined as follows:

$$\left\{ \begin{array}{l} v={\dot{q}}_{d}-\Lambda e\\ a=\dot{v}={\ddot{q}}_{d}-\Lambda \dot{e}\\ r=\dot{q}-v=\dot{e}+\Lambda e \end{array}\right..$$

Another simplified form of the control input can be obtained by linearizing the parameters of Equation (4):

(8) $$u=Y(q,\dot{q},a,v)\hat{\theta }+Z(q)\hat{\pi }-Kr,$$

where the functions ***Y*** and ***Z*** are the regressors of the first three terms and the fourth terms on the left side of Equation (4). $\hat{\theta }$ and $\hat{\pi }$ are the parameter vectors of the corresponding estimated model (two control inputs). Substituting Equation (8) into Equation (4) and linearizing the parameters, the designed closed-loop system equation can be obtained:

(9) $$M(q)\dot{r}+C(q,\dot{q})r+Kr=Y(\hat{\theta }-\theta )+Z(\hat{\pi }-\pi ).\;$$

As mentioned above, considering the uncertainty of model parameters, the following design is made:

(10) $$\hat{\theta }=\theta_{0}+\delta \theta ;\;\hat{\pi }=\pi_{0}+\delta \pi ,\;$$

where ***θ***_0_ and ***π***_0_ are the constant vectors of the corresponding parameter vectors (the theoretical calculation values); *δ**θ*** and *δ**π*** are two design control terms used for compensating the disturbance caused by uncertainty. For the above uncertainty (the difference between the actual value and the calculated value), it can be expressed as:

(11) $$\left\|\tilde{\theta }\right\|=\left\|\theta -\theta_{0}\right\|\leq \rho ;\;\left\|\tilde{\pi }\right\|=\left\|\pi -\pi_{0}\right\|\leq \sigma ,\;$$

where $\tilde{\theta }$ represents the parameter uncertainty of the dynamic model and $\tilde{\pi }$ represents the uncertainty of the link length and the interaction force. Selecting the upper bound constants *σ* and *ρ*. The designs of *δ**θ*** and *δ**π*** are as follows:

(12) $$\delta \theta =\left\{ \begin{array}{l} -\rho \frac{Y^{T}r}{\left\|Y^{T}r\right\|}\;\;\;\;\;\left\|Y^{T}r\right\|>\varepsilon \\ -\rho \frac{Y^{T}r}{\varepsilon }\;\;\;\;\;\;\;\left\|Y^{T}r\right\|\leq \varepsilon \end{array}\right.,\;$$

(13) $$\delta \pi =\left\{ \begin{array}{l} -\sigma \frac{Z^{T}r}{\left\|Z^{T}r\right\|}\;\;\;\;\;\left\|Z^{T}r\right\|>\eta \\ -\sigma \frac{Z^{T}r}{\eta }\;\;\;\;\;\;\;\left\|Z^{T}r\right\|\leq \eta \end{array}\right.,\;$$

where *ε* and *η* are two positive constants used to ensure the continuity of the design term.

In order to analyze the stability of the designed closed-loop system by the Lyapunov second method, the following Lyapunov function is selected:

(14) $$V=\frac{1}{2}r^{T}M(q)r+e^{T}\Lambda Ke.\;$$

Taking the derivative of Equation (14) along the system (9):

(15) $$\dot{V}=-{\dot{e}}^{T}K\dot{e}-e^{T}\Lambda^{T}K\Lambda e+r^{T}Y(\tilde{\theta }+\delta \theta )+r^{T}Z(\tilde{\pi }+\delta \pi ).\;$$

According to the Lyapunov second method, if a Lyapunov function derivative along the system direction is strictly negative definite, it can be determined that the system is asymptotically stable. No matter what state the system starts from, the error will eventually converge to zero. However, in order to ensure Equation (15) is negative definite, additional constraints need to be found. First, rewrite Equation (15) into the following form:

(16) $$\dot{V}=-A^{T}QA+r^{T}Y(\tilde{\theta }+\delta \theta )+r^{T}Z(\tilde{\pi }+\delta \pi ),\;$$

where $A^{T}=[e^{T},\;{\dot{e}}^{T}]$, $Q=\mathrm{diag}[\Lambda^{T}K\Lambda ,\;K]$. Although the first term of Equation (16) can be determined to be semi-negative definite, there are four possible combinations of the last two terms. Since the structures of these two items are similar, the last item is used as an example for analysis. First, when $\left\|Z^{T}r\right\|>\eta$, according to the Cauchy-Schwartz inequality we can obtain:

(17) $${\left(Z^{T}r\right)}^{T}(\tilde{\pi }+\delta \pi )={\left(Z^{T}r\right)}^{T}(\tilde{\pi }-\sigma \frac{Z^{T}r}{\left\|Z^{T}r\right\|})\leq \left\|Z^{T}r\right\|(\left\|\tilde{\pi }\right\|-\sigma )<0.\;$$

When $\left\|Z^{T}r\right\|\leq \eta$, we can be obtained:

(18) $${\left(Z^{T}r\right)}^{T}(\tilde{\pi }+\delta \pi )\leq {\left(Z^{T}r\right)}^{T}(\sigma \frac{Z^{T}r}{\left\|Z^{T}r\right\|}-\sigma \frac{Z^{T}r}{\eta })=-\frac{\sigma }{\eta }{\left\|Z^{T}r\right\|}^{2}+\sigma \left\|Z^{T}r\right\|.\;$$

When the designed item is in the state of Equation (17), the judgment condition is satisfied. When the design item is in the state of Equation (18), Equation (18) can be regarded as a quadratic function about $\left\|Z^{T}r\right\|$. Its maximum value ση/2 at $\left\|Z^{T}r\right\|=\eta /2$ can be obtained, and then the conditions for guaranteeing the Equation (15) is negative definite can be obtained.

According to the designed terms *δ**θ*** and *δ**π***, two maximum values ση/2 and ρε/2 can be obtained respectively. It is not difficult to find that if $A^{T}QA$ is always greater than the sum of these two maximum values, Equation (16) is less than zero forever in all cases (four combinations). In other words, when Equation (19) is satisfied:

(19) $$A^{T}QA>(\sigma \eta +\rho \varepsilon )/2.\;$$

Using the matrix eigenvalue relation $A^{T}QA\geq \lambda_{\min}{\left\|A\right\|}^{2}$ (where *λ*_min_ is the minimum eigenvalue of the matrix ***Q***), the constraints that guarantee Equation (15) is negative definite could be obtained:

(20) $$\left\|A\right\|>{\left[(\sigma \eta +\rho \varepsilon )/2\lambda_{\min}\right]}^{1/2}.\;$$

When Equation (20) is satisfied, $\dot{V}$ can be guaranteed to be less than zero. Therefore, according to Lyapunov second method, the tracking error of system (9) under the designed control law is uniformly ultimately bounded. That is to say, selecting appropriate coefficients in Equations (12) and (13) can ensure that the error continuously approaches a sufficiently small upper error bound, and a good tracking performance could be obtained.

## 4. Experiment

In order to verify the function, feasibility and effectiveness of the proposed lower limb flexion and extension strength training, eight stroke survivors were selected to participate in the test experiment using LLR-II. Every subject confirmed the protocol of the experiment, and research was carried out following the principles of the Declaration of Helsinki. All experiments were conducted under the premise of ensuring the subject’s safety, and sufficient time was given to familiarize the subjects with LLR-II before the formal experiment. The training trajectory is a straight line passing through the hip joint and parallel to the ground, and its starting point and length are determined according to the user’s leg length and knee joint rotation range. The knee joint angle range of all training trajectories in experiments was consistent. Due to the height difference of subjects, the horizontal position coordinates of the training trajectory are also different. For normalized analysis, the horizontal position in this part is represented by percentage of total track length. 

**Figure 7:**
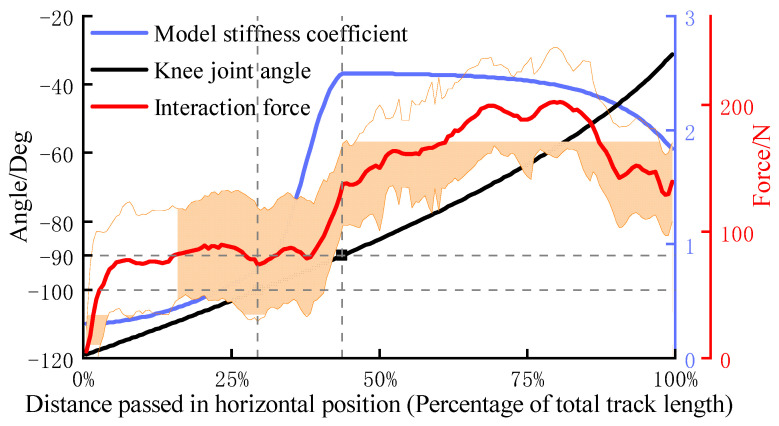
Performance testing of admittance control.

To test the controller performance on guiding users to generate the force, the training interaction force was recorded through the six-dimensional force sensor. In the experiment, each subject was required to maintain higher training speed in three groups of training. Figure 7 shows the changes in knee joint angle *q*_2_, model stiffness ***K***_var_ and effective interaction force *F*_x_ during training. In the experiment, the adjustment constant coefficients *k*_i_ (i = 1, 2 …) of ***K***_var_ are set to 0.3, 0.8, 2.5, 1.5, 6. The average of the end interaction force was calculated, and error bars were plotted based on its standard deviation, as the red line and the orange area shown in the figure. With the stiffness change based on the knee joint angle, the interaction force also displayed a similar trend. Although the strength levels of different subjects were inconsistent, the data results show that the controller has achieved the function of guiding the user to make forces. 

**Figure 8:**
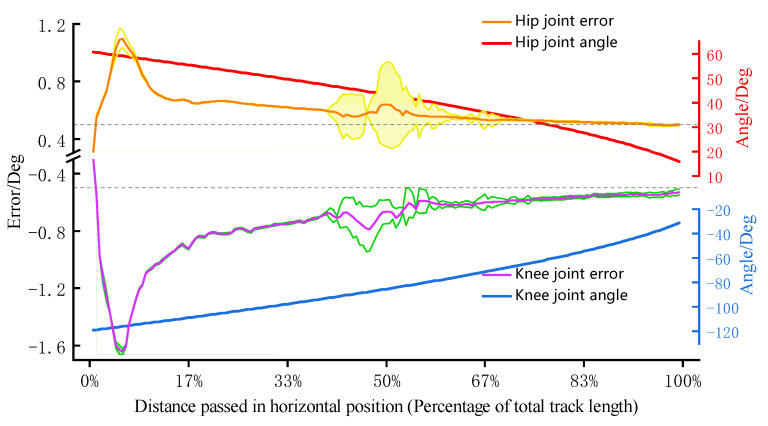
Joint tracking performance.

To analyze the tracking performance of the dual input robust controller, joint angle data in training were recorded as shown in Figure 8. Observing average error curves, it can be found that the absolute values of each joint steady-state error are close to about 0.5°. The result shows that the controller has good tracking ability, and it is in line with the final boundedness proved before. Moreover, it can be found that the two joint errors (orange and purple lines) and error bars (yellow and green areas) appear to be fluctuations in the half of the trajectory. The maximum standard deviation of the hip joint is 0.29°, while the knee joint is 0.16°. This is due to the rapid force increase when the subject tries to adapt to the model stiffness change. The interaction force influence is different to two joints, but the controller can make adjustments to adapt to different sudden interference. It shows that the designed robust controller has strong robustness.

**Figure 9:**
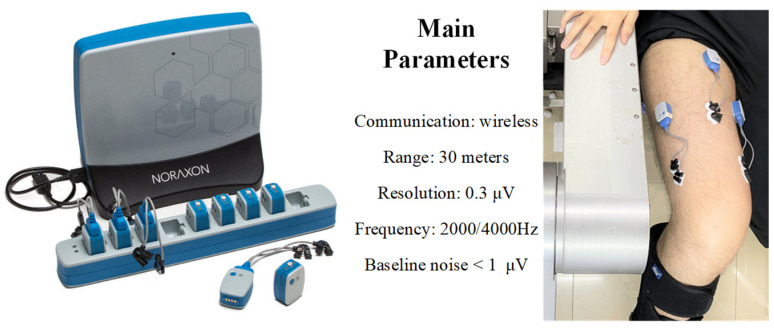
Parameters of EMG device and electrode patch positions.

The EMG signal is a physiological indicator that can directly reflect neuromuscular activity [37,38,39]. This experiment verifies the effectiveness of this strength training by collecting the quadriceps EMG signal during training. The quadriceps femoris is divided into rectus femoris, vastus medialis, vastus lateralis and vastus intermedius. Since the vastus intermedius is located in the deep part of the muscle group, only the EMG signals of the other three muscles were collected in this experiment. EMG device information and electrode patch positions are shown in Figure 9. The positions of the electrode patches are selected under the doctor’s guidance.

**Figure 10:**
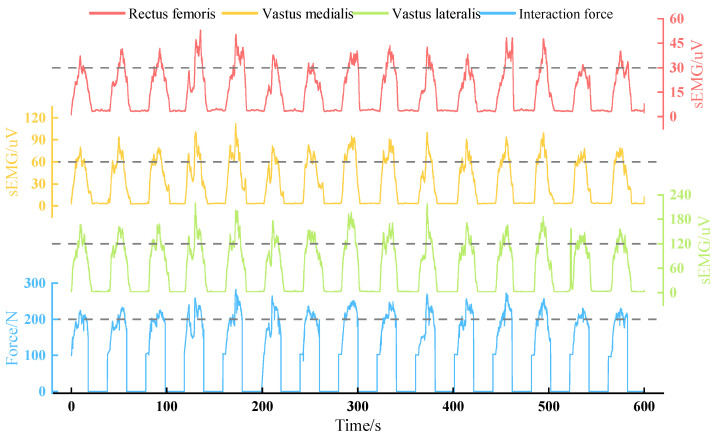
Changes of EMG and interaction force during the training (Dashed line for value reference).

Figure 10 shows the changes in the EMG signals and terminal interaction force of each muscle during a 10-min training. In order to extract the features of EMG signals, the original data was processed by high and low-pass filtering, absolute value taking and smoothing, respectively. The interaction force collected by the force sensor was also plotted in the figure. It can be found that all the data in the training action area are significantly higher. 

## 5. Discussion

The model stiffness of the admittance controller can be adjusted in real-time according to the knee joint angle during training, thereby avoiding excessive compressive force on the knee joint soft tissue and enhancing the muscle stimulation. Through the result of the controller performance experiment, it can be found that the model stiffness ***K***_var_ changes strictly depending on the knee joint angle according to the designed function during training, as the blue line shown in Figure 7. Under the effect of stiffness adjustment, the subject only needs about 90 N to maintain the target training speed when the knee joint flexes more than 90°. This shows that this controller successfully guides users to avoid making forces in the posture that soft tissue is the main bearing object. Meanwhile, the quadriceps femoris enter the most active area when the knee extends to about 90°, and all subjects reported that the training speed at this stage was significantly slowed down. This is due to the change in stiffness, which led to the reduction of the model output acceleration. In order to maintain the training speed, the average interaction force of subjects can reach around 200 N. The results above show that designing a variable stiffness admittance model can indirectly control the terminal interaction force, and it can reduce the possibility of joint soft tissue injury and enhance exertion force in the effective training range. Moreover, the designed robust admittance control can ensure joint tracking performance even under the strong influence of interaction force, and it makes the robot meet the task requirements of this active strength training. In experiments, the peak value of the terminal interaction force was basically above 200 N. The EMG peak value of the vastus lateralis muscle was around 150 uV; the peak value of the vastus medialis muscle was around 75 uV; the peak value of the rectus femoris muscle is around 45 uV. The signal performance of these muscles is consistent with the results of related lower body training studies [33], and obvious signal increase means that the quadriceps femoris is in an active state. These prove that the target muscle group has received effective stimulations under this active strength training method.

There are some limitations in this study and they need to be further studied. Firstly, we design a robust admittance controller strategy to guide users to generate the force correctly during training, and it can be regarded as a method of avoiding soft tissue bearing too much stress under the biomechanics theory support. However, we are unable to provide accurate data on the reduction of soft tissue stress or the actual contribution of this method so far. We planned to conduct a controlled experiment, but it may put control group subjects at risk of injury. We believe that we still need to find a non-invasive method for measuring joint stress to provide strong proof for our work. On the other hand, we selected eight stroke survivors for testing under the recommendation of doctors, and we obtained the result that this training provides effective stimulation to the target muscle group through the sEMG information. Obviously, the sample data are not enough to conclude stronger results, and we ignored to study of the intervention of this training to different types of stroke survivors. Although we believe that the training efficiency can be increased (compared to other same type training) by guiding users to generate forces intensively in an efficient range, there is still a lack of clinical trial data that can quantify the rehabilitation effect of this strength training. We have to recognize that the work shown in the paper is still preliminary research, and more testing experiments need to be carried out later.

We think the rehabilitation robot research should not only consider the training effect but ignore the potential hidden dangers, especially for the elderly group of stroke survivors. In the robot-assist rehabilitation field, few researchers have focused on knee joint protection. This research presents a solution as an attempt to this research gap, but its clinical effect needs a long-term follow-up observation. However, this research still proposes a new point to robot-assisted training: potential negative factors should be considered in order to provide better rehabilitation medical devices for the elderly. 

## 6. Conclusions

In order to avoid an excessive compressive force on the joint soft tissues and increase the stimulation to the target muscle during the leg flexion and extension training, this paper proposes a human–robot cooperative lower limb active strength training based on a robust admittance control strategy. The robust admittance control strategy mainly includes variable stiffness admittance control and dual input robust control. The variation law of admittance model stiffness is designed according to the knee joint biomechanics. The main controller can adjust the stiffness of the model in real-time according to the angle of the knee joint and indirectly control the exertion force of users; the subsidiary admittance control can increase the training flexibility and compliance. Dual input robust control can improve joint tracking performance under the influence of the disturbance caused by the model uncertainty, interactive forces, and external noise. The experiment results show that the designed controller can effectively reduce the possibility of joint soft tissue injury and enhance the stimulation of the quadriceps, and this active training method is effective for exercising the quadriceps. In order to evaluate the efficacy of this strategy, it will be applied to more clinical experiments in future works. 

## Data Availability

Not applicable.

## References

[1] Feng Y., Wang H., Du Y., Chen F., Yan H., Yu H. (2017). Trajectory planning of a novel lower limb rehabilitation robot for stroke patient passive training. Adv. Mech. Eng..

[2] Feng Y., Wang H., Lu T., Vladareanuv V., Li Q., Zhao C. (2017). Teaching training method of a lower limb rehabilitation robot. Int. J. Adv. Rob. Syst..

[3] Johnson C.O., Nguyen M., Roth G.A., Nichols E., Alam T., Abate D., Abd-Allah F., Abdelalim A., Abraha H.N., Abu-Rmeileh N.M. (2019). Global, regional, and national burden of stroke, 1990–2016: A systematic analysis for the global burden of disease study 2016. Lancet Neurol..

[4] Ochi M., Wada F., Saeki S., Hachisuka K. (2015). Gait training in subacute non-ambulatory stroke patients using a full weight-bearing gait-assistance robot: A prospective, randomized, open, blinded-endpoint trial. J. Neurol. Sci..

[5] Maciejasz P., Eschweiler J., Gerlach-Hahn K., Jansen-Troy A., Leonhardt S. (2014). A survey on robotic devices for upper limb rehabilitation. J. Neuroeng. Rehabil..

[6] Wang H., Feng Y., Yu H., Wang Z., Vladareanuv V., Du Y. (2018). Mechanical design and trajectory planning of a lower limb rehabilitation robot with a variable workspace. Int. J. Adv. Rob. Syst..

[7] Colombo R., Pisano F., Micera S., Mazzone A., Delconte C., Carrozza M., Dario P., Minuco G. (2005). Robotic techniques for upper limb evaluation and rehabilitation of stroke patients. IEEE Trans. Neural Syst. Rehabil. Eng..

[8] Mazzoleni S., Puzzolante L., Zollo L., Dario P., Posteraro F. (2014). Mechanisms of motor recovery in chronic and subacute stroke patients following a robot-aided training. IEEE Trans. Haptics.

[9] Aprile I., Iacovelli C., Goffredo M., Cruciani A., Galli M., Simbolotti C., Pecchioli C., Padua L., Galafate D., Pournajaf S. (2019). Efficacy of end-effector robot-assisted gait training in subacute stroke patients: Clinical and gait outcomes from a pilot bi-centre study. NeuroRehabilitation.

[10] Tole G., Raymond M.J., Williams G., Clark R.A., Holland A.E. (2022). Strength training to improve walking after stroke: How physiotherapist, patient and workplace factors influence exercise prescription. Physiother. Theory Pract..

[11] Liu Y., Li X.L., Zhu A., Zheng Z., Zhu H. (2021). Design and evaluation of a surface electromyography-controlled lightweight upper arm exoskeleton rehabilitation robot. Int. J. Adv. Rob. Syst..

[12] Khadanga S., Savage P.D., Ades P.A. (2019). Resistance Training for Older Adults in Cardiac Rehabilitation. Clin. Geriatr. Med..

[13] Kristensen J., Burgess S. (2013). A comparison of two 3-week resistance training programmes commonly used in short-term military rehabilitation. J. R. Army Med. Corps.

[14] Halley D., Paul Ewing B.M. (2013). The X-10: A revolution in knee rehabilitation. Reconstr. Rev..

[15] Wannaphan P., Chanthasopeephan P. (2016). Position controlled knee rehabilitation orthotic device for patients after total knee replacement arthroplasty. Iop Conf..

[16] Yoshioka T., Sugaya H., Kubota S., Onishi M., Kanamori A., Sankai Y., Yamazaki M. (2016). Knee-extension training with a single-joint hybrid assistive limb during the early postoperative period after total knee arthroplasty in a patient with osteoarthritis. Case Rep. Orthop..

[17] Fukaya T., Mutsuzaki H., Yoshikawa K., Sano A., Mizukami M., Yamazaki M. (2017). The training effect of early intervention with a hybrid assistive limb after total knee arthroplasty. Case Rep. Orthop..

[18] Olinski M., Gronowicz A., Handke A., Ceccarelli M. (2016). Design and characterization of a novel knee articulation mechanism. Int. J. Appl. Mech. Eng..

[19] Riener R., Lunenburger L., Jezernik S., Anderschitz M., Colombo G., Dietz V. (2005). Patient-cooperative strategies for robot-aided treadmill training: First experimental results. IEEE Trans. Neural Syst. Rehabil. Eng..

[20] Wu Q., Wang X., Chen B., Wu H. (2018). Development of a minimal-intervention-based admittance control strategy for upper extremity rehabilitation exoskeleton. IEEE Trans. Syst. Man Cybern.-Syst..

[21] Aguirre-Ollinger G., Colgate J.E., Peshkin M.A., Goswami A. (2011). Design of an active one-degree-of-freedom lower-limb exoskeleton with inertia compensation. Int. J. Rob. Res..

[22] Meuleman J., van Asseldonk E., van Oort G., Rietman H., van der Kooij H. (2016). LOPES II-design and evaluation of an admittance controlled gait training robot with shadow-leg approach. IEEE Trans. Neural Syst. Rehabil. Eng..

[23] dos Santos W.M., Siqueira A.A.G. (2019). Optimal impedance via model predictive control for robot-aided rehabilitation. Control Eng. Pract..

[24] Rosado W.M.A., Ortega A.B., Valdes L.G.V., Ascencio J.R., Beltrán C. (2017). Active rehabilitation exercises with a parallel structure ankle rehabilitation prototype. IEEE Lat. Am. Trans..

[25] Zhang F., Hou Z.G., Cheng L., Wang W., Chen Y., Hu J., Peng L., Wang H. (2016). iLeg—A lower limb rehabilitation robot: A proof of concept. IEEE Trans. Hum.-Mach. Syst..

[26] Huang Y., Song R., Argha A., Savkin A.V., Celler B.G., Su S.W. (2020). Continuous description of human 3D motion intent through switching mechanism. IEEE Trans. Neural Syst. Rehabil. Eng..

[27] Li M., Liang Z., He B., Zhao C.-G., Yao W., Xu G., Xie J., Cui L. (2019). Attention-controlled assistive wrist rehabilitation using a low-cost EEG sensor. IEEE Sens. J..

[28] Rouse C.A., Downey R.J., Gregory C.M., Cousin C.A., Duenas V.H., Dixon W.E. (2020). FES Cycling in Stroke: Novel Closed-Loop Algorithm Accommodates Differences in Functional Impairments. IEEE Trans. Biomed. Eng..

[29] Reininga I.H.F., Stevens M., Wagenmakers R., Bulstra S.K., Akker-Scheek I.V.D. (2012). Minimally invasive total hip and knee arthroplasty-implications for the elderly patient. Clin. Geriatr. Med..

[30] Leslie M. (2000). Knee osteoarthritis management therapies. Pain Manag. Nurs. Off. J. Am. Soc. Pain Manag. Nurses.

[31] Nisell R., Ekholm J. (1986). Joint load during the parallel squat in powerlifting and force analysis of in vivo bilateral quadriceps tendon rupture. Scand. J. Sports Sci..

[32] Li G., Zayontz S., Most E., DeFrate L.E., Suggs J.F., Rubash H.E. (2004). In situ forces of the anterior and posterior cruciate ligaments in high knee flexion: An in vitro investigation. J. Orthop. Res..

[33] Escamilla R.F. (2001). Knee biomechanics of the dynamic squat exercise. Med. Sci. Sports Exerc..

[34] Escamilla R.F., Fleisig G.S., Zheng N., Barrentine S.W., Wilk K.E., Andrews J.R. (1998). Biomechanics of the knee during closed kinetic chain and open kinetic chain exercises. Med. Sci. Sports Exerc..

[35] Yan H., Wang H., Vladareanu L., Lin M., Vladareanu V., Li Y. (2019). Detection of participation and training task difficulty applied to the multi-sensor systems of rehabilitation robots. Sensors.

[36] Feng Y., Wang H., Vladareanu L., Chen Z., Jin D. (2019). New Motion Intention Acquisition Method of Lower Limb Rehabilitation Robot Based on Static Torque Sensors. Sensors.

[37] Marin C., Marti M.J., Tolosa E., Alvarez R., Montserrat L., Santamaria J. (1995). Muscle activity changes in spasmodic torticollis after botulinum toxin treatment. Eur. J. Neurol..

[38] Falla D., Jull G., O’Leary S., Dall’Alba P. (2006). Further evaluation of an EMG technique for assessment of the deep cervical flexor muscles. J. Electromyogr. Kinesiol..

[39] Okubo Y., Kaneoka K., Imai A., Shiina I., Tatsumura M., Izumi S., Miyakawa S. (2010). Comparison of the activities of the deep trunk muscles measured using intramuscular and surface electromyography. J. Mech. Med. Biol..

